# Women convicted for violent offenses: Adverse childhood experiences, low level of education and poor mental health

**DOI:** 10.1186/1471-244X-9-81

**Published:** 2009-12-22

**Authors:** Astrid Rossegger, Nicole Wetli, Frank Urbaniok, Thomas Elbert, Franca Cortoni, Jérôme Endrass

**Affiliations:** 1 Psychiatric/Psychological Service, Criminal Justice System, Canton of Zurich, Feldstrasse 42, Zurich, 8090, Switzerland; 2 Department of Psychology, University of Konstanz, Universitätsstraße 10, Konstanz, 78464, Germany; 3 École de criminologie, University of Montreal, PO Box 6128, Station Centre-ville, Montréal (Québec), H3C 3J7, Canada

## Abstract

**Background:**

In past years, the female offender population has grown, leading to an increased interest in the characteristics of female offenders. The aim of this study was to assess the prevalence of female violent offending in a Swiss offender population and to compare possible socio-demographic and offense-related gender differences.

**Methods:**

Descriptive and bivariate logistic regression analyses were performed for a representative sample of N = 203 violent offenders convicted in Zurich, Switzerland.

**Results:**

7.9% (N = 16) of the sample were female. Significant gender differences were found: Female offenders were more likely to be married, less educated, to have suffered from adverse childhood experiences and to be in poor mental health. Female violent offending was less heterogeneous than male violent offending, in fact there were only three types of violent offenses females were convicted for in our sample: One third were convicted of murder, one third for arson and only one woman was convicted of a sex offense.

**Conclusions:**

The results of our study point toward a gender-specific theory of female offending, as well as toward the importance of developing models for explaining female criminal behavior, which need to be implemented in treatment plans and intervention strategies regarding female offenders.

## Background

### Female offenders

A substantial increase in female offending, e.g. in England and the United States over the past two decades [1,5,6], has spurred the interest of researchers into learning more about the characteristics of female offenders, the kind of crimes women are more likely to commit, the circumstances which drive women to offending, and the factors that determine the risk for repeat offending (e.g. Steffensmeier [7]).

The origins of female offending have been explored from various perspectives and within different disciplines. The emancipation theory put forth by Adler [8] argues that as the gender roles of women change and become more and more similar to those of men, so will their participation in criminal activity - a theory not supported by criminal statistics. From a different angle, South Richardson and Hammock [9] examined whether the social role men and women have in society influences male and female aggression in connection with criminal behavior. Campbell, Muncer and Bibel [10] took an evolutionary approach and argued that the scarcity of resources drives women to perpetrate crimes. From a cognitive point of view, Bennett, Farrington and Huesmann [2] explained that women have lower rates of offending due to the fact that they have better prosocial skills than men and because they acquire these social cognitive skills earlier on in life, in comparison to their male counterparts. Hollin and Palmer [11] discussed the criminogenic needs of women and whether they are specific to women only.

One of the main questions being asked in the literature is to which extent a gender-neutral approach would suffice and for which areas gender-specific aspects would be necessary in order to create a model for female offending. Many of the researchers point out that the origins of male offending cannot simply be transferred to female offending without necessary investigation as entirely different determinants might be responsible for it [1-3,11,12]. Other researchers argue that these gender-specific theories do not account for the full range of female offending and that gender-neutral theories would suffice to explain both female and male criminality [13,14].

When reviewing the literature on male and female offending, it becomes apparent that there are similarities, as well as differences between the both, regarding the characteristics of the offense, the offender and the victim. It seems that men and women mostly commit the same types of crimes, but that the rates of female offending are substantially lower than male offending across all crime categories [7,13,15]. For both male and female adult offenders, we can see the predominance of property and drug offenses [12,13], but it appears that the gender gap is greatest for violent offenses: Women generally seem to have much lower crime rates for violent offending than men [3,7,15-18]. While some researchers have found an indication towards an increase of female violent offending [4,19] others claim that the rate of female violent crime has remained stable [3,5].

### Female violent offenders

The current literature supports the hypothesis that female violent offenders display a different offense pattern in comparison to male violent offenders. Female violent offending is often characterized by the victims of women often being people close to them, the crime a result of interpersonal conflict - this includes parents, husbands and boyfriends, as well as children [9,10,13,18,20-22]. In comparison to men, women are more likely to commit a violent crime at home. Weizmann-Henelius et al. [18] concluded that female violent behavior more often leads to the death of a close victim than that of an acquaintance or stranger. In a study by Roe-Sepowitz [22], it was documented that females mostly offended violently during the perpetration of another crime, such as robbery. There are, however, some similarities in male and female violent offense patterns: Both men and women commit crimes within the context of an argument and the victim is more likely to be male [20].

Concerning socio-demographic characteristics, both female and male violent offenders were often found to have a history of adverse childhood domestic constellations (absent and/or criminal parents, neglect), poor socio-economic backgrounds, equal rates of previous psychiatric hospitalization, to be less educated, and under- or unemployed at the time of offense [6,7,14,21]. In comparison to male offenders, female offenders were more likely to be older, married and have children. They were also more likely to be presently victims of physical, psychological and sexual abuse, as well as childhood physical, psychological and sexual abuse. They showed also elevated levels of substances abuse, and were more frequently suffering from physical and mental illness [3,5,7,11,18,23]. Yourstone [21] established that female offenders were less likely to be foreign nationals than male offenders. Female offenders have been found to have less prior convictions [5,21], have lower rates of psychopathy (see the Hare Psychopathy-Checklist-Revised [24]) [14,25,26] and are less likely to recidivate than male offenders [7].

The aim of the present study was to assess the prevalence of female violent offending in a representative population of Swiss violent offenders. A second aim was to assess socio-demographic, psychiatric and offense-related characteristics of female violent offending in Switzerland and to compare these female offenders with their male counterparts.

## Methods

### Sample selection criteria

The sample comprised all female and male violent and sex offenders that were convicted between August 2000 and December 2002 in the Canton of Zurich, Switzerland. The Canton of Zurich is an urban, sub-alpine area with a population of 1.2 million inhabitants. The concrete inclusion criterion was: Sentenced to a minimum prison sentence of ten months due to a violent or sex offense, or to court-ordered therapy by a court in the Canton of Zurich between August 2000 and December 2002. Two hundred and three offenders matched these criteria and have been included in the study sample.

### Procedures and measures

Data was collected from correctional and court files by five research assistants, all trained psychologists holding the degree of Master of Science (MSc). There was no direct contact with the offenders. The files contained comprehensive personal details, including criminal history, the exact type and circumstances of the offense, as well as personality characteristics and psychiatric diagnoses (if any). Based on this information, socio-demographic characteristics (e.g. gender, age at the time of the index offense, level of education, marital status), childhood conditions (e.g. sexual and/or physical abuse, living in a foster home), psychiatric characteristics (e.g. diagnoses - if any, previous hospitalization, family history of psychiatric disorders, substance abuse), and offense-related characteristics (e.g. number and type of previous offenses; characteristics of the index offense: relationship to the victim, number of victims, degree of injury of the victim, type of offense and sentence) were collected. The interrater reliability was satisfactory (Kappa > 0.65).

### Statistical analysis

Descriptive and bivariate logistic regression analyses were carried out with STATA 10.0.

### Ethical approval

The sample of the present study is a subsample of a large epidemiological study conducted on convicted offenders (inmates as well as offenders on probation) in the Canton of Zurich in the year 2000, which was approved as a whole by an external Ethics Committee (Kantonale Ethikkommission Zürich). In agreement with the committee, no informed consent needed to be obtained as there was no contact with any of the study subjects. All data was collected entirely from the subjects' files and anonymized before further analysis.

## Results

### Socio-demographic characteristics

With 187 (92%) of the 203 offenders, there were distinctly more male than female (7.9%, N = 16) offenders. The mean age of the sample was 32.5 years (SD = 10.8) with a range from 18 to 65. Male and female offenders did not differ significantly concerning their age (female: 31.5 years, male: 32.7 years).

Fifty-five point five percent (N = 112) of the offenders were Swiss nationals. Female offenders were 3.8 times more likely to be Swiss nationals than male offenders (OR = 3.8, p < .05): Among the female offenders, 81.3% (N = 13) were Swiss and among the male offenders 53.2% (N = 99).

Furthermore, there were differences regarding the civil status at time of the offense: The odds of a female offender being married was 2.9 times higher than for a male offender (OR = 2.89, female: 50%, N = 8; male: 26%, N = 48). Thirty-nine percent (N = 78) of the offenders had at least one child. Male and female offenders did not differ with respect to this criterion.

Female offenders were consistently less likely to finish their school education (OR = 0.18). Only one third (31.3%, N = 5) of the female offenders completed their school education, whereas 72% (N = 126) of the male offenders hold the equivalent of a high school diploma. However, male and female offenders did not differ significantly concerning their vocational education (female: 43.8%, N = 7; male: 51.7%, N = 93).

Table 1 gives an overview of the socio-demographic characteristics of the study sample stratified for gender as well as the results of the bivariate logistic regression analysis using gender as the dependent variable.

**Table 1:** Socio-demographic differences of female and male sex and violent offenders

	All		Male		Female		OR	95% CI	
Swiss national	112	55.5	99	53.2	13	81.3	3.81*	1.05	13.81
Being married	56	27.9	48	26.0	8	50.0	2.89	1.02	8.02
Having a child	78	39.0	71	38.6	7	43.8	1.23	0.44	3.47
At least 9 years of school	131	68.6	126	72.0	5	31.3	0.18*	0.06	0.53
Vocational education	100	51.0	93	51.7	7	43.8	0.73	0.26	2.04


*Note*. OR = Odds Ratio. 95% CI = Confidence Interval.* p < .05.

### Offense-related characteristics

#### Index offense

Between 2000 and 2002 the courts of the Canton of Zurich convicted only one woman for committing a sex offense leading to a sentence of at least 10 months. 37.5% (N = 6) of the convicted female violent offenders committed murder and one third arson (31.3%, N = 5).

Table 2 shows the index offenses stratified for gender.

**Table 2:** Index offense characteristics stratified for gender

		All		Male		Female	
		
		N	%	N	%	N	%
Sex offenses	Child sexual abuse	44	21.7	43	23.0	1	6.3
	Rape	57	28.1	57	30.5	0	-
	Other sex offenses	3	1.5	3	1.6	0	-

Violent offenses	Robbery	27	13.3	25	13.4	2	12.5
	Assault	16	7.9	14	7.5	2	12.5
	Murder	25	12.3	19	10.2	6	37.5
	Abduction	2	1.0	2	1.1	0	-
	Arson	20	9.9	15	8.0	5	31.3
	Endangerment of human life	9	4.4	9	4.8	0	-

	Total	203	100.0	187	100.0	16	100.0


#### Offense pattern

In one out of three index offenses (33.7%, N = 68), the victim was severely or fatally injured. The percentage of severe or fatal injuries was higher for the offenses perpetrated by the female offenders (female: 56.3%, N = 9; male: 31.7%, N = 59). Female offenders tended to assault family members more frequently than male offenders (female: 25.0%, N = 4, male: 10.2%, N = 19) - however, this finding was not significant on a 5% level, which might be due to the small N and the subsequent low statistical power. Female offenders were also more likely to suffer from delusional symptoms at the time of the offense: 20% (N = 3) in the female strata vs. 3.5% in the male strata. Alcohol was involved in one third of the offenses (32.5%, N = 63). Male and female offenders did not differ concerning this characteristic.

#### Criminal history

Fifty-nine point seven percent (N = 120) of the sample had a criminal record. The prevalence of a criminal record did not differ significantly between male and female offenders (female: 46.7%, N = 7; male: 60.8%, N = 113). However, the female offenders were less likely to have a criminal record for repeat offending than men (female: 6.7%, N = 1; male: 19.9%, N = 37; OR = 0.29). 37.5% (N = 6) of the women in our sample had a previous record for prostitution (male offenders: 1.8%, N = 3).

#### Family history and childhood

Women showed a higher level of stressful life events in childhood. Women reported having experienced childhood sexual abuse ten times more often than men (OR = 9.67, p < 0.05). Women furthermore reported more frequently having a family history involving alcohol abuse or dependency (male: 20.3%, N = 27; female: 40.0%, N = 6), as well as a higher rate of growing up in a violent family environment (male: 24.2%, N = 30; female: 46.7%, N = 7), though these differences were not significant on a p < .05 level. 46.7% (N = 7) of the female offenders had been treated in a psychiatric clinic prior to the index offense in comparison to only 24.4% (N = 41) of the male offenders (table 3).

**Table 3:** Gender differences in childhood, health, criminal history and index offense

		All		Male		Female		Logistic regression	
		
		N	%	N	%	N	%	OR	95% CI
Index offense	Victim severely wounded or dead	68	33.7	59	31.7	9	56.3	2.77	0.98-7.79
	Related victim	23	11.4	19	10.2	4	25.0	2.93	0.86-9.99
	Delusional at the time of the offense	9	4.8	6	3.5	3	20.0	7.00*	1.55-31.52
	Alcohol involved in the offense	63	32.5	57	32.0	6	37.5	1.27	0.44-3.68

Criminal history	Criminal record	120	59.7	113	60.8	7	46.7	0.57	0.20-1.63
	Pertinent criminal record	38	18.9	37	19.9	1	6.7	0.29	0.04-2.26

Childhood	Family alcohol abuse	33	22.3	27	20.3	6	40.0	2.62	0.86-7.99
	Sexually abused as a child	9	5.9	5	3.6	4	26.7	9.67*	2.27-41.30
	Violence in the nuclear family	37	26.6	30	24.2	7	46.7	2.74	0.92-8.19

Mental health	Treated in a psychiatric clinic	48	26.2	41	24.4	7	46.7	2.71	0.93-7.93
	Prostitution	9	5.0	3	1.8	6	37.5	32.6*	7.09-149.94


*Note*. OR = Odds Ratio. 95% CI = Confidence Interval. * p < 0.05.

## Discussion

The aim of the present study was to assess the prevalence of female violent offending in a representative population of violent offenders in Switzerland. A second aim was to assess socio-demographic, psychiatric and offense-related characteristics of female violent offending in Switzerland and to compare these female offenders with their male counterparts.

In this representative Swiss sample of N = 203 violent offenders, the proportion of female offenders was 8% and female violent offending was less heterogeneous than male violent offending. If a woman commits a violent offense in the Canton of Zurich, it is quite likely to be homicide or arson.

The literature describes female offenders as frequently having been victims of childhood physical, psychological and sexual abuse [3,5,7,11,18,23]. Also in our study female offenders were at a higher risk of having suffered child hood sexual abuse: females were 10 times more likely to have suffered this type of abuse than their male counterparts. The proportion of women offenders (46.7%) who reported having experienced violence within their families was similarly high. A further result corroborating results of previous studies was the high prevalence of mental health disorders in the population of female offenders [3,5,7,11,18,23]. In the present study, 46.7% of the women had been hospitalized in a psychiatric hospital at least once in the past and 20% were delusional at the time of offense. A further distinct female offender characteristic was prostitution prior to the index offense.

Another result found and discussed in the literature, namely that the victims of women offenders are often people close to them, the crime a result of interpersonal conflicts [9,10,13,18,20-22] could only be corroborated tentatively: One out of four female offenders assaulted a relative - compared to one out of ten for the male offenders. Although not statistically significant, most likely due to the small sample size, women were four times more likely to commit murder than their male counterparts (37.5% vs. 10.2). In accordance with the literature, which states that female violent behavior more often leads to the death of a close victim than of an acquaintance or stranger [18], these results suggest that violent behavior in female offenders is not the result of antisocial personality traits, but rather the result of an interpersonal conflict which resulted in the death of the victim. There was also a tendency for women offenders to have less prior convictions than men, which supports the results of Forsyth, Wooddell and Evans [5] and Yourstone [21].

The analysis of the socio-demographic data reveals that the violent female offenders differed somewhat from their male counterparts: Even though not significant, there seemed to be a tendency for female offenders to be married more frequently than male offenders. To some degree, these results corroborate the findings of other studies which also showed that most female offenders were found to be married [3,5,7,11,18,23]. Corroborating the findings of Yourstone [21], an impressive difference was found concerning nationality; namely 81.3% of the female but only 53.2% of the male offenders were Swiss citizens. However, considering the well known high threshold in becoming a Swiss citizen, overcome easily only by marriage, one wonders whether nationality is a valid variable to distinguish male from female offenders in this study group. Contrary to findings in the literature, our sample of female offenders was less likely to attain a higher level of education and were more likely to be unemployed than male offenders.

## Conclusions

Female offenders definitely differ from male offenders. In summary, a picture emerges showing that female, in comparison to male offenders, were victims of childhood physical, psychological and sexual abuse and that conduct problems were evident early on (lack of graduation). In adulthood, there appears to be a tendency toward poor mental health (psychiatric hospitalization) and prostitution prior to the offense. Violent offenses (homicide and arson) are committed in a delusional state. The victims of female offenders are family members in 25% of the cases. The majority of female offenders have no prior convictions.

Altogether, female offenders often appear to be mentally ill and/or to have problematic personalities. Adversity and traumatic stress - ranging from sexual abuse to emotional neglect - and life stress, especially when present during developmental periods, render the victims prone to mental illness, such as trauma-spectrum disorders (e.g. [27,28]), later depression [29], anxiety disorders [30] and psychosis [31]. Consequently, improved mental health service is necessary for these cases early on. In addition to the beneficiary effects for the patients, it may substantially contribute to the control of their offending behavior.

Whether gender-specific rather than gender-neutral approaches are suitable for explaining female criminality has remained a matter of controversy. Given that violent offenses by women occur in small numbers it is difficult to provide sufficient, reliable and robust information. In addition, there is a social taboo on female violence [18,32], which might result in a certain reluctance to classify women offenders in a particular way.

In our opinion, the results of this study point towards a gender-specific theory of female offending, and we must stress the importance that models explaining female criminal behavior be developed in order to be implemented in treatment plans and intervention strategies regarding female offenders.

## Limitations

Even though the sample is representative for a certain time frame within a local region of Switzerland, the sample population of female offenders remains quite small. The statistical power for differences between male and female offenders was therefore small.

## Competing interests

The authors declare that they have no competing interests.

## Authors' contributions

AR was responsible for the conception of the study, the statistical analysis of the data and interpretation of the results and helped draft the manuscript. NW was responsible for the first draft of the manuscript. FU developed the idea for the study and was responsible for its funding. TE and FC have been involved in revising the manuscript critically. JE was substantially involved in the conception of the study, the analysis of the data, the interpretation of the results and writing the manuscript. All authors read and approved the final manuscript.

## Pre-publication history

The pre-publication history for this paper can be accessed here:

http://www.biomedcentral.com/1471-244X/9/81/prepub
